# Monocyte to HDL cholesterol ratio predicts obesity-associated cardiac dysfunction

**DOI:** 10.7555/JBR.38.20240432

**Published:** 2025-05-28

**Authors:** Chunsheng Zhao, Jinting Liu, Jiaqi Zhao, Chao Wang, Hui Bai, Qing Yang, Jingjing Ben, Xudong Zhu, Xiaoyu Li, Bin Jiang, Kai Li, Runfeng Sun, Xuexing Ma, Liansheng Wang, Hanwen Zhang, Qi Chen

**Affiliations:** 1 Department of Pathophysiology, Nanjing Medical University, Nanjing, Jiangsu 211166, China; 2 Key Laboratory of Jiangsu Province on Targeted Intervention of Cardiovascular Diseases, Nanjing Medical University, Nanjing, Jiangsu 211166, China; 3 Key Lab of Modern Toxicology of Ministry of Education, Center for Global Health, School of Public Health, Nanjing Medical University, Nanjing, Jiangsu 211166, China; 4 Donghai County People's Hospital, Donghai, Jiangsu 222300, China; 5 Department of Cardiology, the Affiliated Suzhou Hospital of Nanjing Medical University, Suzhou, Jiangsu 215006, China; 6 Department of Cardiology, the First Affiliated Hospital of Nanjing Medical University, Nanjing Medical University, Nanjing, Jiangsu 211166, China

**Keywords:** cardiac dysfunction, obesity, monocyte to HDL-C ratio, left ventricular ejection fraction

## Abstract

As the prevalence of obesity increases dramatically, obesity-associated cardiac dysfunction constitutes a considerable challenge to human health. This study aimed to identify more useful lipid/inflammatory markers to predict the risk of obesity-associated cardiac dysfunction. By retrospectively analyzing the clinical characteristics of 5648 cardiac disease patients, we found that both the plasma level of high-density lipoprotein cholesterol (HDL-C) and the blood monocyte count were significantly associated with impairment of the left ventricular ejection fraction (LVEF). Univariate and multivariate regression analyses revealed that the monocyte to HDL-C ratio (MHR) was a more powerful predictor of the risk of LVEF decline than either HDL-C or monocyte alone. Mediation analysis further revealed a mediating effect of a high MHR on the decline in obesity-associated cardiac systolic function. Collectively, our results demonstrate a superior role of MHR in predicting the risk of an obesity-associated decline in cardiac systolic function among routine metabolic/inflammatory markers.

## Introduction

Cardiovascular disease (CVD) remains the leading global cause of mortality, affecting over 500 million individuals worldwide^[1]^. As the population ages and metabolic risk factors escalate, the burden of CVD is projected to rise further^[2]^. Among various CVDs, heart failure (HF) is a syndrome characterized by symptoms and signs of compromised left ventricular function resulting in reduced longevity. The incidence of HF has increased in recent years, with its prevalence in China rising by 44% over the past 15 years, mirroring global trends in CVD-related morbidity and mortality^[3]^.

Heart failure can be caused by multiple conditions, including coronary artery disease, hypertension, cardiomyopathies, valvular and congenital heart disease, and arrhythmias, among which ischemic heart disease is the predominant cause of HF^[4–5]^. The conventional risk factors, such as dyslipidemia, hypertension, and smoking, have shown a gradual decline in recent years. Obesity, lack of exercise, and diabetes are increasing in prevalence worldwide, greatly contributing to the pathogenesis of HF^[6]^. Obesity causes cardiac dysfunction through multiple mechanisms, including chronic low-grade inflammation, myocardial hypertrophy, fibrosis, hemodynamic changes, and metabolic disorders^[7]^. Despite advances in imaging modalities capable of identifying obesity-associated cardiac dysfunction, improved risk prediction for the intervention is needed to guide timely intervention in clinical practice. Especially, accessible and cost-effective biomarkers will facilitate early detection and risk stratification for the disease.

The interplay between dyslipidemia and inflammation contributes to the pathophysiology of obesity-associated cardiac dysfunction, making identification of relevant biomarkers crucial^[8–9]^. Fasting plasma triglycerides (TG), total cholesterol (TC), low-density lipoprotein cholesterol (LDL-C), and high-density lipoprotein cholesterol (HDL-C) are routine markers for the diagnosis of dyslipidemia. Obesity-induced chronic inflammation triggers elevations in white blood cell (WBC) and monocyte (MO) counts, high-sensitivity C-reactive protein (hs-CRP), and inflammatory cytokines such as interleukin 1β (IL-1β), IL-6, and tumor necrosis factor α (TNFα). While measurements of these markers provide an important reference base for the assessment of cardiovascular risk, their predictive consistency for cardiovascular outcomes encounters challenges across diverse populations. For example, ≥ 3 mg/L of plasma hs-CRP is accepted as an inflammatory status in Western populations, whereas 0.9 mg/L is accepted in Japan and Korea, highlighting the need for more appropriate biomarkers^[10]^.

The monocyte to high-density lipoprotein cholesterol (MHR) ratio has emerged as an integrative marker reflecting both the inflammatory burden and lipoprotein metabolic status. An increased MHR value is associated with higher long-term mortality and major adverse cardiovascular events (MACEs) in chronic heart disease patients, which is independent of established risk factors^[11–12]^. MHR serves as an independent predictor of severity, all-cause mortality, and in-hospital complications in patients with coronary artery disease, particularly in those with hypertension^[13–14]^. Correlation of MHR with carotid intima-media thickness has also been documented in patients with type 2 diabetes^[15]^. However, the role of MHR in predicting the risk of obesity-related cardiac dysfunction is unclear.

The present study aimed to explore the association between MHR and cardiac function in heart disease patients. Our study demonstrates that MHR is a reliable marker of obesity-related cardiac dysfunction. Its accessibility and cost-effectiveness make MHR a valuable predictor for the early identification of high-risk individuals, including those in resource-limited or rural settings.

## Materials and methods

### Study population and ethical considerations

**Figure 1:**
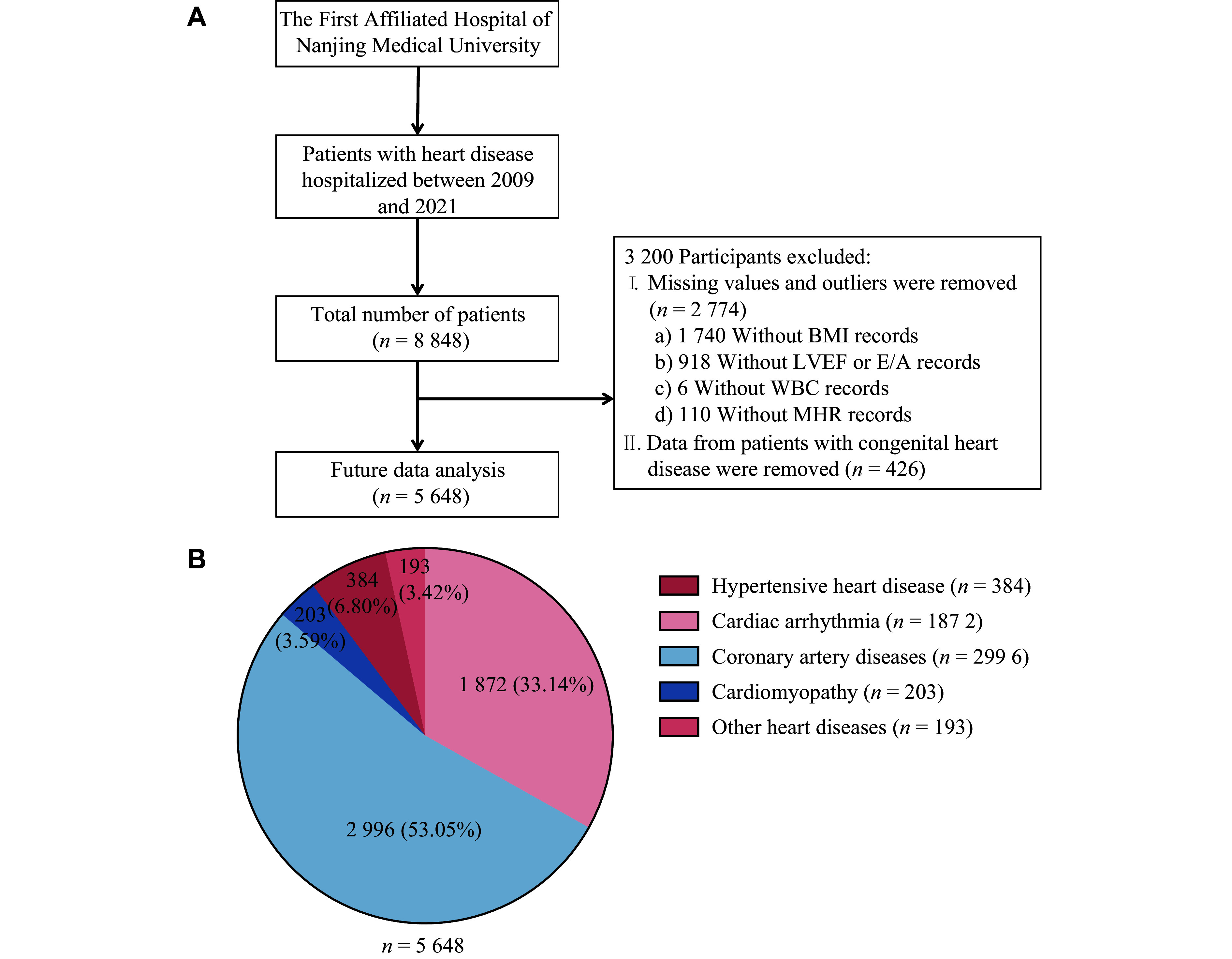
Study flowchart and disease spectrum. A: Flowchart of selecting studied participants. In total, 8848 patients with primary cardiac disease hospitalized in the Department of Cardiology, the First Affiliated Hospital of Nanjing Medical University between 2009 and 2021 were recruited. After excluding individuals with incomplete data and patients with congenital heart disease, a population of 5648 subjects was finally included in the study. B: Pie chart of diagnosed cardiovascular disease types. Data are presented as *n* (%).

This retrospective cross-sectional study was conducted on a cohort of 8848 patients diagnosed with primary cardiac diseases, including coronary heart disease, hypertension, arrhythmias, and cardiomyopathies, who were hospitalized at the Department of Cardiology, the First Affiliated Hospital of Nanjing Medical University, between 2009 and 2021 (***Fig. 1***). The patient population encompassed a wide range of socioeconomic backgrounds, from public service employees to agricultural workers. Patients with congenital or genetic heart diseases, active infections, severe hepatic or renal dysfunction, and acute exacerbations of chronic diseases during the study period, as well as those with incomplete data, were excluded. Specific exclusions included 426 patients diagnosed with congenital heart disease, 1740 patients without height or weight data, 918 patients without ventricular early diastolic function or E/A ratio (the ratio of early to late diastolic mitral inflow velocities) measurements, six patients without peripheral WBC data, and 110 patients without MHR data. After exclusions, a final cohort of 5648 patients was included in the analysis. This study was approved by the Institutional Review Board of Nanjing Medical University (Approval No. NMU-2024-192), and all participants provided written informed consent in accordance with the Declaration of Helsinki.

### Data collection

Demographic and clinical data, including age, sex, height, weight, smoking habits, alcohol consumption, and the presence of chronic metabolic conditions (hypertension and diabetes), were systematically recorded by trained clinicians at admission. Body height and weight were measured while participants stood upright, wearing light clothing and no shoes. After participants fasted for 12 h, blood samples were collected in anticoagulant vacuum tubes and centrifuged to isolate plasma. Plasma levels of glucose (FPG), TG, TC, LDL-C, and HDL-C were measured using an Olympus AU640 autoanalyzer (Olympus, Kobe, Japan). Regular calibration of laboratory equipment and blinded sample analysis were employed to maintain data integrity and minimize measurement bias.

Cardiac function was assessed *via* echocardiography using the GE LOGIQ E9 system. Left ventricular end-diastolic diameter and end-systolic diameter were measured, and ventricular volumes, including end-diastolic volume and end-systolic volume, were calculated using the ventricular area method^[16]^. Left ventricular ejection fraction (LVEF) was derived using the Teichholz method, and real-time flow monitoring was conducted *via* echocardiography. Pulsed Doppler imaging was used to analyze mitral flow, assessing early (E) and late (A) diastolic velocities to calculate the E/A ratio^[17]^.

### Data definitions and classification criteria

Dyslipidemia was defined based on the following criteria: (1) hypercholesterolemia, plasma TC ≥ 5.2 mmol/L; (2) hypertriglyceridemia, TG ≥ 1.7 mmol/L; (3) elevated LDL-C, ≥ 3.4 mmol/L; (4) reduced HDL-C, < 1.03 mmol/L for males or < 1.29 mmol/L for females^[18]^. Body mass index (BMI) classification was based on the standards set by the Centers for Disease Control and Prevention (CDC) of the USA and the World Health Organization (WHO). A healthy BMI was defined as 18.5–24.9 kg/m^2^, overweight as 25.0–30.0 kg/m^2^, and obesity as ≥ 30.0 kg/m^2[19–20]^. Plasma levels of hs-CRP ranging from zero to 10 mg/L and blood WBC counts from four to 10 × 10^9^/L were defined as normal^[21]^. The MHR was calculated as the ratio of monocyte count to HDL-C level^[22]^.

## Statistical analysis

Categorical variables were summarized as frequencies and percentages. All continuous variables were confirmed to have non-normal distributions using the Kolmogorov-Smirnov test and Q-Q plot, and were expressed as medians with quartiles. Categorical variables were compared using the Chi-square (*χ*^2^) test, and continuous variables were compared using the Kruskal–Wallis H test for multiple-group comparisons. To explore the determinants of cardiac systolic and diastolic functions, univariate and multivariate linear regression analyses were performed. Collinearity diagnostics identified multicollinearity between TC and LDL-C, necessitating the use of bidirectional stepwise regression in multivariate models. Factors such as age, sex, smoking, alcohol consumption, hypertension, and diabetes were adjusted as confounders.

The relationship between BMI and LVEF was analyzed using generalized additive models (GAMs), with model smoothness optimized *via* generalized cross-validation^[23]^. Mediation analyses were employed to evaluate the mediating role of MHR in the association between elevated BMI and reduced LVEF. The predictive performance of the biomarkers was assessed using receiver operating characteristic (ROC) curves, with diagnostic accuracy quantified by the area under the curve (AUC). Differences between the ROC curves of different biomarkers and baseline were statistically compared using the DeLong nonparametric test. Optimal cutoff values were determined by maximizing the Youden index, defined as the sum of sensitivity and specificity minus one (Youden index = sensitivity + specificity − 1). All statistical analyses were conducted using IBM SPSS Statistics (version 26.0) and R statistical software (version R-3.6.1). Mediation analyses were carried out using the PROCESS v2.16.3 plug-in in SPSS. A two-sided *P*-value < 0.05 was considered statistically significant^[24]^.

## Results

### Obese patients with heart disease with lower HDL-C levels and higher blood monocyte numbers suffer from more severe cardiac dysfunction

The clinical characteristics of the study cohort are summarized in ***Table 1***. The cohort consisted primarily of middle-aged and older adults, with a median age of 60.5 years. Males accounted for 59.19% (3343) and females for 40.81% (2305) of the participants. In addition to demographic data, lifestyle habits and chronic metabolic conditions were also analyzed. We found that 20.02% (1131) of the patients were long-term smokers, 13.93% (787) consumed alcohol regularly, 6.55% (370) had a history of hypertension, and 9.47% (535) had a history of diabetes.

**Table 1:** The baseline demographic and clinical characteristics of 5648 patients with heart disease

Characteristics	Total (*n* = 5648)	BMI ≤ 18.50 (*n* = 63)	18.5 < BMI ≤ 24.9 (*n* = 1746)	25.0 ≤ BMI ≤ 30.0 (*n* = 2490)	BMI ≥ 30.0 (*n* = 1349)	*H*/*χ*^2^	*P*
Demographic							
Sex [*n* (%)]						160.303	< 0.001
Male	3343 (59.19)	20 (31.75)	841 (48.17)	1576 (63.29)	906 (67.16)		
Female	2305 (40.81)	43 (68.25)	905 (51.83)	914 (36.71)	443 (32.84)		
Age [years, M (Q1, Q3)]	60.50 (51.00, 69.00)	52.00 (27.00, 75.00)	60.00 (51.00, 69.00)	61.00 (52.00, 69.00)	58.00 (48.00, 67.00)	30.099	< 0.001
Height [cm, M (Q1, Q3)]	167.00 (160.00, 172.00)	165.00 (160.00, 177.00)	165.00 (160.00, 170.00)	168.00 (160.00, 172.00)	168.00 (160.00, 173.00)	67.486	< 0.001
Weight [kg, M (Q1, Q3)]	70.00 (62.00, 79.00)	46.00 (44.00, 51.00)	60.00 (55.00, 65.00)	71.00 (66.00, 77.00)	84.00 (77.00, 90.00)	3180.497	< 0.001
Smoker [*n* (%)]	1131 (20.02)	13 (20.63)	374 (21.42)	499 (20.04)	245 (18.16)	5.063	0.167
Alcohol drinker [*n* (%)]	787 (13.93)	10 (15.87)	240 (13.75)	360 (14.46)	177 (13.12)	1.563	0.668
Hypertension [*n* (%)]	370 (6.55)	7 (11.11)	90 (5.15)	136 (5.46)	137 (10.16)	41.159	< 0.001
Diabetes [*n* (%)]	535 (9.47)	1 (1.59)	125 (7.16)	244 (9.80)	165 (12.23)	27.747	< 0.001
TG [mmol/L, M (Q1, Q3)]	1.29 (0.95, 1.86)	1.20 (0.80, 1.50)	0.90 (0.70, 1.30)	0.80 (0.70, 1.10)	0.80 (0.70, 1.10)	415.994	< 0.001
TC [mmol/L, M (Q1, Q3)]	3.96 (3.30, 4.68)	3.78 (3.18, 4.54)	4.03 (3.36, 4.71)	3.92 (3.25, 4.63)	4.00 (3.30, 4.73)	10.141	0.017
HDL-C [mmol/L, M (Q1, Q3)]	1.02 (0.88, 1.20)	1.24 (1.03, 1.39)	1.12 (0.94, 1.31)	1.00 (0.87, 1.17)	0.97 ( 0.85, 1.11)	310.073	< 0.001
LDL-C [mmol/L, M (Q1, Q3)]	2.31 (1.84, 2.84)	2.10 (1.69, 2.52)	2.30 (1.86, 2.84)	2.28 (1.81, 2.82)	2.37 (1.87, 2.89)	14.494	0.002
PLT [10^9^/L, M (Q1, Q3)]	189.00 (156.00, 228.00)	197.00 (151.00, 245.00)	187.00 (154.00, 227.25)	189.00 (156.00, 226.00)	192.00 (159.50, 234.00)	11.302	0.010
ALT [U/L, M (Q1, Q3)]	21.50 (14.60, 32.90)	14.20 (9.90, 18.80)	18.80 (12.50, 28.50)	22.30 (15.20, 32.70)	25.50 (17.80, 38.70)	278.472	< 0.001
AST [U/L, M (Q1, Q3)]	21.70 (17.90, 27.40)	20.10 (16.20, 24.60)	20.90 (17.10, 26.30)	22.30 (15.20, 32.70)	22.90 (18.70, 29.60)	66.699	< 0.001
Glu [mmol/L, M (Q1, Q3)]	4.83 (4.38, 5.51)	4.39 (3.98, 4.72)	4.65 (4.26, 5.27)	4.85 (4.43, 5.56)	5.01 (4.54, 5.85)	160.256	< 0.001
Cr [mmol/L, M (Q1, Q3)]	69.80 (58.88, 81.30)	63.10 (50.80, 75.00)	66.10 (56.78, 78.20)	71.10 (60.30, 82.40)	71.90 (60.50, 83.30)	102.712	< 0.001
Left ventricular structure and function							
LAD [mm, M (Q1, Q3)]	36.00 (33.00, 39.00)	30.00 (27.00, 34.00)	34.00 (31.00, 37.00)	37.00 (34.00, 40.00)	38.00 (35.00, 41.00)	709.158	< 0.001
LVDd [mm, M (Q1, Q3)]	47.00 (45.00, 50.00)	43.00 (40.00, 47.00)	46.00 (43.00, 49.00)	48.00 (45.00, 50.00)	48.00 (46.00, 51.00)	365.619	< 0.001
IVS [mm, M (Q1, Q3)]	10.00 (10.00, 11.00)	9.00 (8.00, 10.00)	10.00 (9.00, 10.00)	10.00 (10.00, 11.00)	10.00 (10.00, 11.00)	440.293	< 0.001
E/A [M (Q1, Q3)]	0.80 (0.70, 1.20)	1.20 (0.80, 1.50)	0.90 (0.70, 1.10)	0.80 (0.70, 1.10)	0.80 (0.70, 1.10)	79.781	< 0.001
EF [%, M (Q1, Q3)]	63 (61, 65)	65 (64, 66)	64 (62, 66)	63 (61, 64)	63 (61, 64)	410.353	< 0.001
Inflammation							
hs-CRP [mg/L, M (Q1, Q3)]	3.02 (1.47, 5.78)	2.32 (1.08, 5.00)	3.02 (1.44, 5.72)	3.02 (1.53, 6.18)	3.02 (1.45, 5.08)	8.267	0.041
WBC [10^9^/L, M (Q1, Q3)]	6.11 (4.59, 9.00)	5.69 (4.00, 8.00)	6.00 (4.55, 9.06)	6.26 (4.70,9.49)	6.08 (4.50, 8.62)	6.884	0.076
MO [10^9^/L, M (Q1, Q3)]	0.47 (0.38, 0.59)	0.38 (0.33, 0.53)	0.44 (0.36, 0.55)	0.48 (0.39, 0.59)	0.49 (0.40, 0.61)	104.049	< 0.001
MHR [M (Q1, Q3)]	0.46 (0.34, 0.62)	0.35 (0.24, 0.44)	0.40 (0.29, 0.54)	0.48 (0.36, 0.64)	0.51 (0.40, 0.67)	286.370	< 0.001
Data are presented as *n* (%) or median and the first and third quartiles [M (Q1, Q3)], and analyzed using the Kruskal–Wallis test or Pearson's Chi-square test. Abbreviations: ALT, alanine aminotransferase; AST, aspartate aminotransferase; Cr, creatinine; Glu, glucose; E/A, early/atrial peak; EF, ejection fraction; HDL-C, high-density lipoprotein cholesterol; hs-CRP, high-sensitivity C-reactive protein; IVS, interventricular septum; LAD, left atrium diameter; LDL-C, low-density lipoprotein cholesterol; LVDd, left ventricular diastolic dimension; MHR, monocyte to high-density lipoprotein cholesterol ratio; MO, monocyte; PLT, platelet; TC, total cholesterol; TG, triglyceride; WBC, white blood cells.							

When participants were categorized into four groups based on BMI, all investigated parameters, except smoking and alcohol consumption, showed statistically significant differences. Compared with participants with lower BMI, those with higher BMI had significantly lower plasma HDL-C levels, higher blood monocyte counts, and MHR values. In terms of cardiac function, the higher BMI groups exhibited lower LVEF and E/A ratios, indicative of reduced systolic and diastolic cardiac function.

### Metabolic disorders and inflammation are associated with the decline in cardiac systolic function

We next performed univariate and multivariate linear regression analyses to assess the associations of metabolic and inflammatory parameters with LVEF and E/A. Multicollinearity was identified between TC and LDL-C (variance inflation factor [VIF] > 10), and bidirectional stepwise regression was therefore applied. For LVEF, significant negative associations were found with diabetes, HDL-C, and LDL-C. Inflammatory markers such as monocyte and MHR, instead of WBC and hs-CRP, also had negative associations with LVEF (***Table 2***). Consistent associations were observed between the E/A ratio and both hypertension and TC. However, parameters such as monocyte count and MHR did not show significant associations with diastolic function (***Table 3***). These findings suggest that cardiac systolic and diastolic functions are differentially associated with the metabolic and inflammatory factors in the studied patients, with hypertension being the predominant factor associated with cardiac diastolic dysfunction. Taken together, elevated blood pressure, diabetes, hypercholesterolemia, and inflammation may be associated with the decline in cardiac systolic function.

**Table 2:** Univariate and multivariate linear regression analysis of the association between LVEF and metabolic/inflammatory markers

Variables	Univariate analysis						Multivariate analysis				
	B	SE	*t*	*P*	*β* (95% CI)		B	SE	*t*	*P*	*β* (95% CI)
Female					0 (Reference)						0 (Reference)
Male	−0.02	0.00	−8.28	< 0.001	−0.02 (−0.02–−0.01)		−0.01	0.00	−2.63	0.008	−0.01 (−0.99–−0.01)
Age (years)	−0.01	0.00	−2.25	0.025	−0.01 (−0.99–−0.01)						
Smoker	0.00	0.00	−0.83	0.405	−0.00 (−0.01–0.00)						
Alcohol drinker	0.00	0.00	−1.05	0.294	−0.00 (−0.01–0.00)						
Hypertension	0.01	0.00	2.75	0.006	0.01 (0.01–0.02)		0.01	0.00	2.59	0.010	0.01 (0.01–0.02)
Diabetes	−0.02	0.00	−5.63	< 0.001	−0.02 (−0.02–−0.01)		−0.01	0.00	−2.80	0.005	−0.01 (−0.01–−0.01)
TG (mmol/L)	0.00	0.00	−0.63	0.532	−0.00 (−0.00–0.00)						
TC (mmol/L)	0.00	0.00	0.94	0.346	0.00 (−0.00–0.00)		0.01	0.00	2.71	0.007	0.01 (0.01–0.02)
HDL-C (mmol/L)	0.03	0.00	9.21	< 0.001	0.03 (0.03–0.04)		−0.01	0.01	−2.03	0.043	−0.01 (−0.03–−0.01)
LDL-C (mmol/L)	0.00	0.00	−1.31	0.190	−0.00 (−0.00–0.00)		−0.02	0.00	−3.72	< 0.001	−0.02 (−0.02–−0.01)
PLT (10^9^/L)	0.01	0.00	2.02	0.043	0.01 (0.01–0.01)		0.01	0.00	3.31	< 0.001	0.01 (0.01–0.01)
ALT (U/L)	−0.01	0.00	−5.43	< 0.001	−0.01 (−0.99–−0.01)						
AST (U/L)	−0.01	0.00	−5.40	< 0.001	−0.01 (−0.99–−0.01)		−0.01	0.00	−3.24	0.001	−0.01 (−0.99–−0.01)
Plasma glucose (mmol/L)	−0.01	0.00	−5.48	< 0.001	−0.01 (−0.99–−0.01)						
Cr (mmol/L)	−0.01	0.00	−10.75	< 0.001	−0.01 (−0.99–−0.01)		−0.01	0.00	−8.35	< 0.001	−0.01 (−0.99–−0.01)
BMI (kg/m^2^)	−0.01	0.00	−9.34	< 0.001	−0.01 (−0.99–−0.01)		−0.01	0.00	−2.88	0.004	−0.01 (−0.99–−0.01)
BMI (kg/m^2^)											
< 18.5					0 (Reference)						0 (Reference)
18.5–24.9	−0.02	0.01	−2.46	0.014	−0.02 (−0.04–−0.01)		−0.01	0.01	−1.04	0.298	−0.01 (−0.03–0.01)
25–29.9	−0.04	0.01	−4.56	< 0.001	−0.04 (−0.06–−0.02)		−0.02	0.01	−1.69	0.091	−0.02 (−0.04–0.00)
≥ 30	−0.04	0.01	−4.51	< 0.001	−0.04 (−0.06–−0.02)		−0.01	0.01	−0.73	0.463	−0.01 (−0.03–0.01)
Inflammation											
hs-CRP (mg/L)	−0.01	0.00	−2.21	0.027	−0.01 (−0.99–−0.01)						
WBC (10^9^/L)	−0.00	0.00	−0.40	0.692	−0.00 (−0.00–0.00)						
MO (10^9^/L)	−0.05	0.00	−12.83	< 0.001	−0.05 (−0.05–−0.04)		−0.07	0.01	−5.09	< 0.001	−0.07 (−0.09–−0.04)
MHR	−0.05	0.00	−12.83	< 0.001	−0.05 (−0.05–−0.04)		−0.07	0.01	−5.09	< 0.001	−0.07 (−0.09–−0.05)
Abbreviations: ALT, alanine aminotransferase; AST, aspartate aminotransferase; BMI, body mass index; CI, confidence interval; Cr, creatinine; HDL-C, high-density lipoprotein cholesterol; hs-CRP, high-sensitivity C-reactive protein; LDL-C, low-density lipoprotein cholesterol; MHR, monocyte to high-density lipoprotein cholesterol ratio; MO, monocyte; SE, standard error; TC, total cholesterol; TG, triglyceride; PLT, platelet; WBC, white blood cells.											

**Table 3:** Univariate and multivariate linear regression analysis of the association between E/A ratio and metabolic/inflammatory parameters

Variables	Univariate analysis						Multivariate analysis				
	B	SE	*t*	*P*	*β* (95% CI)		B	SE	*t*	*P*	*β* (95% CI)
Female					0 (Reference)						0 (Reference)
Male	−0.01	0.01	−0.85	0.397	−0.01 (−0.03–0.01)		−0.04	0.01	−3.01	0.003	−0.04 (−0.06–−0.01)
Age (years)	−0.01	0.00	−31.44	< 0.001	−0.01 (−0.01–−0.01)		−0.01	0.00	−31.89	< 0.001	−0.01 (−0.01–−0.01)
Smoker	0.03	0.01	2.13	0.033	0.03 (0.01–0.06)		0.03	0.01	2.28	0.023	0.03 (0.01–0.06)
Alcohol drinker	0.02	0.02	0.94	0.345	0.02 (−0.02–0.05)						
Hypertension	−0.08	0.02	−3.39	< 0.001	−0.08 (−0.13–−0.03)		−0.13	0.02	−6.16	< 0.001	−0.13 (−0.18–−0.09)
Diabetes	−0.12	0.02	−5.92	< 0.001	−0.12 (−0.16–−0.08)						
TG (mmol/L)	−0.01	0.00	−3.04	0.002	−0.01 (−0.01–−0.01)						
TC (mmol/L)	0.00	0.01	−0.22	0.827	−0.00 (−0.01–0.01)		−0.07	0.02	−3.69	< 0.001	−0.07 (−0.11–−0.03)
HDL-C (mmol/L)	0.06	0.02	2.50	0.012	0.06 (0.01–0.10)		0.09	0.03	3.13	0.002	0.09 (0.04–0.15)
LDL-C (mmol/L)	0.01	0.01	0.71	0.475	0.01 (−0.01–0.02)		0.07	0.03	2.80	0.005	0.07 (0.02–0.12)
PLT (10^9^/L)	0.00	0.00	1.63	0.104	0.00 (−0.00–0.00)		−0.01	0.00	−4.60	< 0.001	−0.01 (−0.99–−0.01)
ALT (U/L)	0.00	0.00	1.02	0.308	0.00 (−0.00–0.00)		0.00	0.00	−1.91	0.056	−0.00 (−0.00–0.00)
AST (U/L)	0.00	0.00	1.51	0.130	0.00 (−0.00–0.00)		0.01	0.00	2.37	0.018	0.01 (0.01–0.01)
Plasma glucose (mmol/L)	−0.03	0.00	−8.81	< 0.001	−0.03 (−0.03–−0.02)		−0.01	0.00	−4.71	< 0.001	−0.01 (−0.02–−0.01)
Cr (mmol/L)	0.00	0.00	−0.61	0.540	−0.00 (−0.00–0.00)		0.01	0.00	3.08	0.002	0.01 (0.01–0.01)
BMI (kg/m^2^)	−0.01	0.00	−7.58	< 0.001	−0.01 (−0.02–−0.01)		−0.01	0.00	−2.70	0.007	−0.01 (−0.02–−0.01)
BMI (kg/m^2^)											
<18.5					0 (Reference)						0 (Reference)
18.5–24.9	−0.16	0.06	−2.94	0.003	−0.16 (−0.27–−0.05)		0.01	0.05	0.21	0.834	0.01 (−0.09–0.12)
25–29.9	−0.26	0.06	−4.73	< 0.001	−0.26 (−0.37–−0.15)		−0.02	0.06	−0.30	0.764	−0.02 (−0.13–0.10)
≥ 30	−0.26	0.06	−4.61	< 0.001	−0.26 (−0.37–−0.15)		0.01	0.07	0.21	0.833	0.01 (−0.12–0.15)
Inflammation											
hs-CRP (mg/L)	0.00	0.00	−0.44	0.663	−0.00 (−0.00–0.00)						
WBC (10^9^/L)	0.00	0.00	−0.60	0.546	−0.00 (−0.00–0.00)						
MO (10^9^/L)	−0.04	0.03	−1.31	0.190	−0.04 (−0.10–0.02)						
MHR	−0.04	0.02	−1.53	0.126	−0.04 (−0.08–0.01)		0.05	0.03	1.76	0.079	0.05 (−0.01–0.10)
Abbreviations: ALT, alanine aminotransferase; AST, aspartate aminotransferase; BMI, body mass index; CI, confidence interval; Cr, creatinine; HDL-C, high-density lipoprotein cholesterol; hs-CRP, high-sensitivity C-reactive protein; LDL-C, low-density lipoprotein cholesterol; MHR, monocyte to high-density lipoprotein cholesterol ratio; MO, monocyte; SE, standard error; TC, total cholesterol; TG, triglyceride; PLT, platelet; WBC, white blood cells.											

### BMI is adversely associated with cardiac function in normal-weight heart disease patients

**Figure 2:**
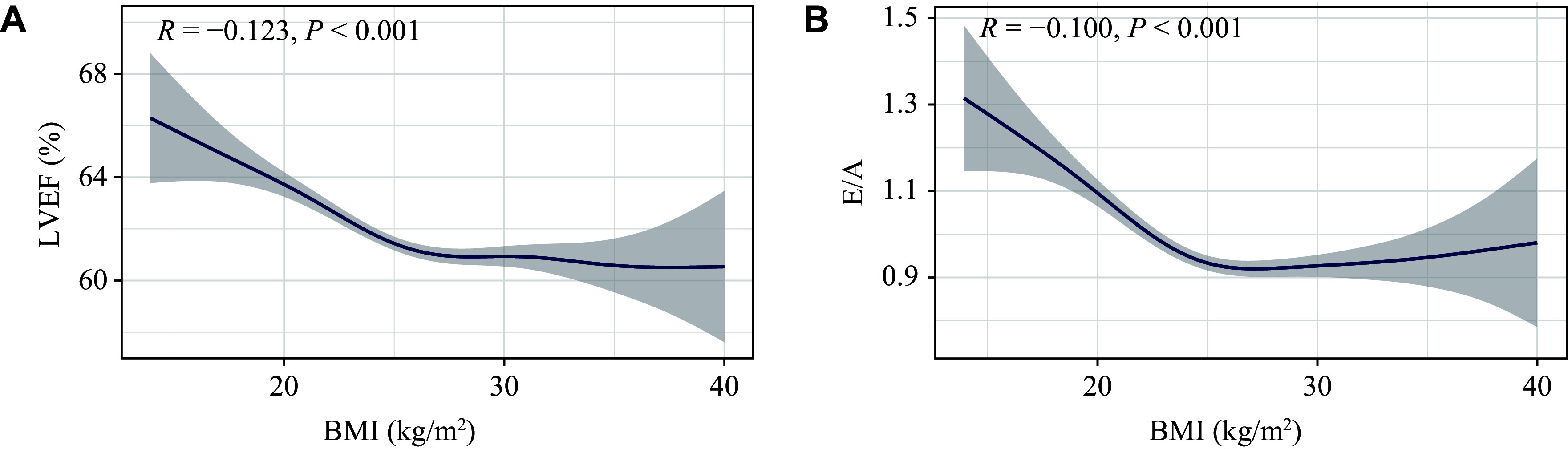
Trends of LVEF and E/A ratio across BMI under the generalized additive model. A: The relationship between LVEF and BMI in 5648 patients with heart disease. Associated *R* and *P *values are shown; shaded portions of the figure indicate 95% CI. B: The relationship between E/A and BMI in 5648 patients with heart disease. Associated *R* and *P* values are shown; shaded portions of the figure indicate 95% CI. Abbreviations: BMI, body mass index; CI, confidence interval; E/A, early/atrial peak; LVEF, left ventricular ejection fraction.

To further explore the influence of obesity on cardiac function, we used a generalized additive model to plot LVEF and E/A ratio trends across various BMI levels (***Fig. 2***). We found that LVEF decreased along with the elevation of BMI when BMI was ≤ 25.0 kg/m^2^. For the E/A ratio, it declined sharply when BMI was ≤ 25.0 kg/m^2^, and increased slightly when BMI > 25.0 kg/m^2^, although the E/A ratio remained below 1.0. These results demonstrate that BMI is adversely associated with cardiac function in heart disease patients within the normal-weight range.

### MHR is a superior predictor of systolic decline in obesity-related cardiac dysfunction

**Figure 3:**
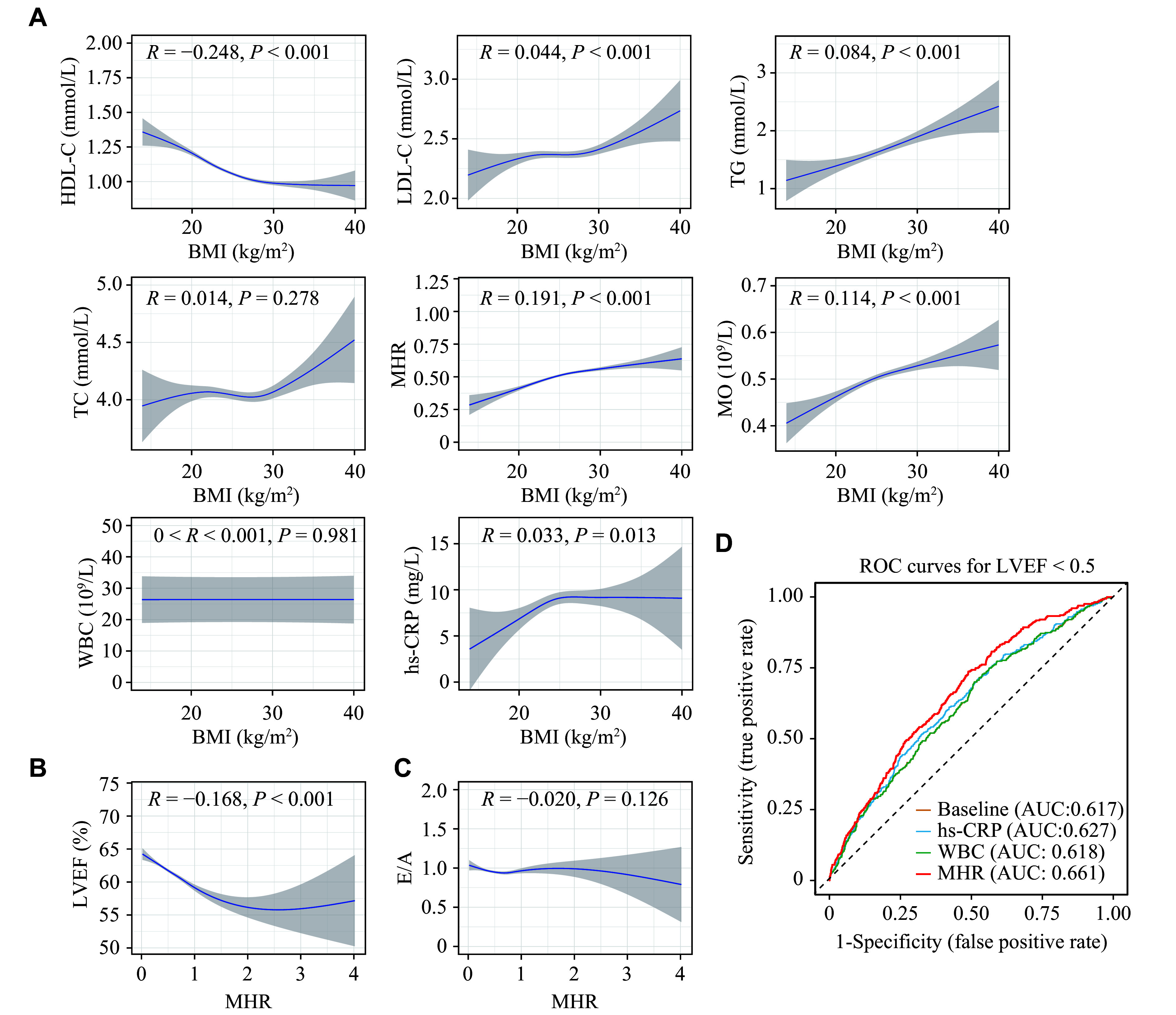
Correlation between BMI, cardiac function, metabolic, and inflammatory parameters. A: Correlation between HDL-C, LDL-C, TG, TC, MHR, MO, WBC, hs-CRP and BMI in 5648 patients with heart disease. B: Correlation between LVEF and MHR in 5648 patients with heart disease. C: Correlation between E/A ratio and MHR in 5648 patients with heart disease. Associated *R* and *P* values are shown; shaded portions of the figure indicate 95% CI. D: Receiver operating characteristic (ROC) curves comparing diagnostic factors for LVEF impairment. Every line represents the diagnostic capability of a specific factor (yellow: baseline; blue: hs-CRP; green: WBC; red: MHR). These factors were analyzed after adjusting for age, sex, smoking, alcohol consumption, hypertension, diabetes, and BMI, which are known risk factors or confounding factors for LVEF impairment. The red line illustrates the improved diagnostic performance achieved when common factors are combined with MHR, yielding a superior area under the curve (AUC) of 0.661, suggesting enhanced diagnostic accuracy. The dashed line represents the line of no discrimination, equivalent to random chance. A DeLong's test of these three correlated ROC curves against the baseline yields a more significant *P*-value of MHR (*P* < 0.001), compared to hs-CRP (*P* = 0.029) and WBC (*P* = 0.460). Abbreviations: BMI, body mass index; CI, confidence interval; E/A, early/atrial peak; HDL-C, high-density lipoprotein cholesterol; hs-CRP, high-sensitivity C-reactive protein; LDL-C, low-density lipoprotein cholesterol; LVEF, left ventricular ejection fraction; MHR, monocyte to high-density lipoprotein cholesterol ratio; MO, monocyte; TC, total cholesterol; TG, triglyceride; WBC, white blood cells.

Considering that obesity-associated cardiac dysfunction involves complex mechanisms, we further used generalized additive models to examine the trends of lipids and inflammatory markers in obesity. Both HDL-C and monocytes showed significant correlations with BMI, and their correlation coefficients were notably higher than those for LDL-C and TG. In contrast, no significant correlations were observed for TC or WBC with BMI (***Fig. 3A***). Further exploration revealed that both HDL-C and monocytes were significantly associated with LVEF, whereas no significant association was found between monocytes and E/A (***Supplementary Figs. 1*** and ***2***). Of note, MHR showed a positive correlation with BMI, and its association with obesity was comparable to that of HDL-C and monocytes (***Fig. 3A***). However, the coefficient between MHR and LVEF increased from 0.12 (for either HDL-C or monocytes) to 0.17, which was not observed in its association with E/A (***Fig. 3B*** and ***3C***, and ***Supplementary Fig. 1***). By comparing the performance of MHR with traditional biomarkers such as WBC count and hs-CRP in predicting the LVEF impairment, we found that the diagnostic sensitivity of MHR (AUC = 0.661) for obesity-induced cardiac dysfunction was significantly higher than that of WBC (AUC = 0.618) and hs-CRP (AUC = 0.627) (***Fig. 3D*** and ***Table 4***). Therefore, these results suggest that MHR may act as a stronger predictor of the decline of systolic function.

**Table 5:** Mediation effect of inflammatory biomarkers on LVEF decline caused by elevated BMI

Mediator	Sample	Exposure to mediator	Mediator to outcome	Direct effect	Mediated (indirect effect)	Total effect (exposure to outcome)	Proportion mediated (%)
MHR	5648	0.0099 (0.0009) *P* < 0.001	−0.0355 (0.0039) *P* < 0.001	−0.0004 (0.0003) *P* < 0.001	−0.0018 (95%CI: −0.0005, −0.0002)	−0.0022 (0.0003) *P* < 0.001	18.18%
MO	5648	0.0040 (0.0007) *P* < 0.001	−0.0326 (0.0050) *P* < 0.001	−0.0021 (0.0003) *P* < 0.001	−0.0001 (95%CI: −0.0002, −0.0001)	−0.0022 (0.0003) *P* < 0.001	4.54%
HDL-C	5648	−0.0152 (0.0009) *P* < 0.001	0.0186 (0.0038) *P* < 0.001	−0.0019 (0.0003) *P* < 0.001	−0.0003 (95%CI: −0.0004, −0.0002)	−0.0022 (0.0003) *P* < 0.001	13.64%
hs-CRP	5648	0.3770 (0.0735) *P* < 0.001	−0.0002 (0.0000) *P* < 0.001	−0.0021 (0.0003) *P* < 0.001	−0.0001 (95%CI: −0.0001, 0.0000)	−0.0022 (0.0003) *P* < 0.001	4.54%
MHR (male)	3343	0.0123 (0.0014) *P* < 0.001	−0.0310 (0.0051) *P* < 0.001	−0.0023 (0.0004) *P* < 0.001	−0.0004 (95%CI: −0.0006, −0.0002)	−0.0027 (0.0004) *P* < 0.001	14.81%
MHR (female)	2305	0.0064 (0.0011) *P* < 0.001	−0.0458 (0.0068) *P* < 0.001	−0.0012 (0.0003) *P* < 0.001	−0.0003 (95%CI: −0.0005, −0.0001)	−0.0015 (0.0003) *P* < 0.001	20.00%
Abbreviations: BMI, body mass index; HDL-C, high-density lipoprotein cholesterol; hs-CRP, high-sensitivity C-reactive protein; LVEF, left ventricular ejection fraction; MHR, monocyte to high-density lipoprotein cholesterol ratio; MO, monocyte.							

**Figure 4:**
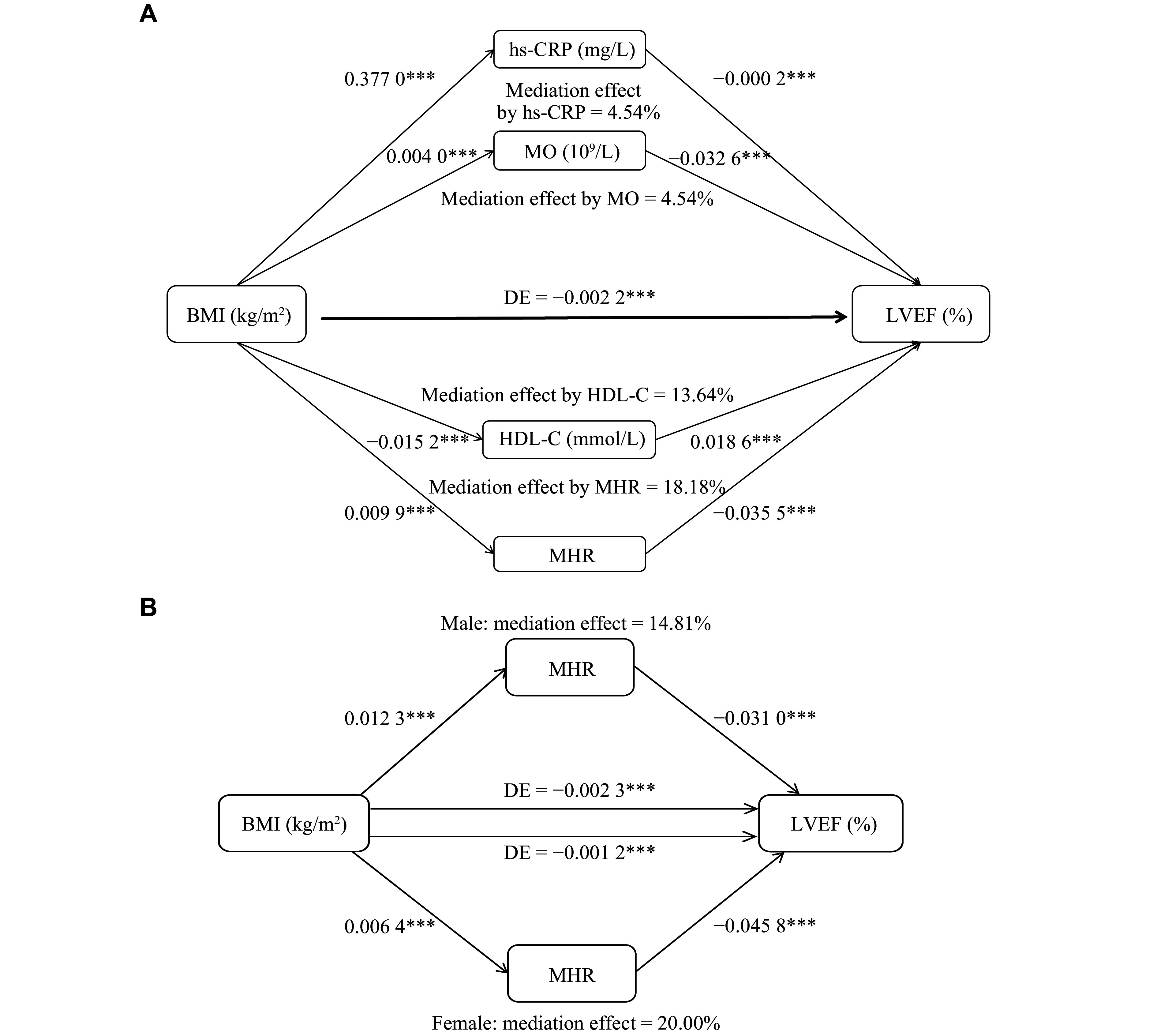
Mediation analysis of BMI elevation and LVEF decline. A: Percentage of mediating effects of elevated BMI on LVEF decline under different mediating measures. ^***^P < 0.001. B: MHR as a mediator in the LVEF decline caused by elevated BMI, comparison between male and female subgroups. ^***^P < 0.001. Abbreviations: BMI, body mass index; DE, direct effect; LVEF, left ventricular ejection fraction; HDL-C, high-density lipoprotein cholesterol; hs-CRP, high-sensitivity C-reactive protein; MHR, monocyte to high-density lipoprotein cholesterol ratio; MO, monocyte.

Finally, we verified the role of MHR in obesity-associated cardiac systolic function through a mediation analysis. After adjusting for confounders such as age, sex, smoking, alcohol consumption, hypertension, and diabetes, we found that the direct effect of BMI on LVEF was −0.0022 (*P* < 0.001). The mediation effect of MHR was the highest (18.18%), significantly higher than those of hs-CRP (4.54%), monocytes (4.54%), and HDL-C (13.64%) (***Fig. 4A*** and ***Table 5***). No significant mediation effect was observed for WBC or other lipid markers (TC, TG, LDL-C). Interestingly, the direct effect of BMI on LVEF was greater in males (−0.0023) than in females (−0.0012), while the mediation effect of MHR was more pronounced in females (20.00%) than in males (14.81%) (***Fig. 4A*** and ***4B***). As such, our findings highlight a superior role of MHR in predicting obesity-associated decline in cardiac systolic function among routine metabolic and inflammatory markers.

**Table 4:** ROC curve analyses of diagnostic factors for LVEF impairment

Model	AUC statistics		Model comparison *P-*value		Optimal cutoff	
	AUC (SE)	95% CI		Cut-off	Sensitivity	Specificity
Baseline	0.617 (0.016)	0.587–0.648	–	0.06	70.00%	48.90%
hs-CRP	0.627 (0.016)	0.597–0.658	0.029	0.07	51.70%	67.40%
WBC	0.618 (0.016)	0.587–0.648	0.460	0.06	70.00%	48.90%
MHR	0.661 (0.015)	0.633–0.690	< 0.001	0.05	73.70%	51.10%
Cutoff determined by Youden index.Abbreviations: AUC, area under the curve; CI, confidence interval; LVEF, left ventricular ejection fraction; hs-CRP, high-sensitivity C-reactive protein; MHR, monocyte to high-density lipoprotein cholesterol ratio; ROC, receiver operating characteristic; SE, standard error; WBC, white blood cells.						

## Discussion

Lipid metabolic disorders and inflammation contribute to the pathogenesis of obesity-associated cardiac dysfunction^[25]^. Abnormalities in lipid profiles, including plasma levels of TC, TG, LDL-C, and HDL-C, have long been recognized as important risk factors that impair cardiovascular health, particularly in the development of coronary heart disease^[26]^. Chronic low-grade inflammation acts as a key driver of many heart diseases, including heart failure. Its biomarkers, such as WBC or monocyte count, hs-CRP, IL-6, IL-1β, and TNF-α, have been shown to independently predict adverse cardiovascular events and disease progression^[27–28]^. However, the risk factors predicting obesity-associated cardiac dysfunction remain unclear. By retrospectively analyzing the data of cardiovascular disease patients, we observed independent associations of low HDL-C plasma levels and high blood monocyte counts with LVEF decline, respectively. Moreover, we demonstrated for the first time that high MHR may serve as a predictive factor for the prognosis of obesity-associated cardiac dysfunction by integrating lipid and inflammatory biomarkers.

Obesity has a well-established association with the development of CVD, including heart failure, coronary heart disease, atrial fibrillation, and hypertension^[29–30]^. Our observation indicates that the adverse association between BMI and cardiac function only occurs in heart disease patients with a healthy body weight but not in overweight patients. This finding may be explained by the phenomenon of the obesity paradox, wherein overweight or mildly obese patients exhibit better survival rates and cardiac functions than their normal-weight counterparts in certain clinical contexts^[31–32]^. Therefore, the identification of novel markers beyond body weight is an unmet need. For example, HDL exerts protective effects against cardiovascular diseases. Elevated HDL levels are associated with a reduced risk of cardiovascular events. Besides inhibiting cholesterol accumulation in cells, suppressing inflammatory responses, and reducing oxidative stress, higher HDL levels correlate with a lower incidence of adverse cardiac outcomes such as fibrosis and ventricular dilation^[33]^. These key features of HDL may explain why the level of HDL is the only lipid marker that predicts cardiac systolic function and obesity in our studied population. Moreover, elevated levels of reactive oxygen species in obesity can oxidize HDL particles, converting them into dysfunctional forms that contribute to endothelial dysfunction and vascular inflammation^[34]^. Obesity also induces HDL particle compositional changes in triglycerides and proteins, leading to the impairment of their cardiovascular protective function^[35]^. Whether assessing the particle heterogeneity of HDL would better predict the risk of obesity-associated cardiac dysfunction warrants further investigation.

In the context of obesity-related cardiovascular diseases, inflammation is thought to be a central pathogenic mechanism through the interactions between inflammatory cytokines and various parenchymal cells^[36]^. Our results revealed that the blood monocyte count had stronger predictive power for the decline in cardiac systolic function than for diastolic function, and performed better than the WBC count and the hs-CRP level. This may be partly attributed to the infiltration of monocytes and macrophages in metabolic organs in obesity and the dominant role of monocytes/macrophages in obesity-induced chronic inflammation^[37]^. Traditional specialized biomarkers may not fully capture the complexity of obesity-induced lipid metabolism disorders and abnormalities in immune regulation. For example, dysfunctional HDL, particularly in the presence of chronic inflammation, can impair HDL properties such as antioxidation, anti-inflammation, cholesterol efflux promotion, and atherosclerosis prevention, even in cases where HDL levels are normal^[38]^. Whether dyslipidemia affects fluctuations in inflammatory markers warrants further investigation. Our findings suggest that MHR outperforms traditional markers like hs-CRP and WBC in predicting obesity-associated cardiac dysfunction. Compared with other emerging inflammatory indices such as the neutrophil-to-lymphocyte ratio^[39]^, MHR is likely superior since monocyte-derived macrophages dominate the obesity-induced chronic inflammation. MHR combines monocyte count with HDL-C levels, providing an integrative window to view inflammation and lipid metabolic disorder in the body. Elevated MHR, reflecting amplified detrimental inflammation and reduced protective HDL-C, is directly linked to higher cardiovascular risk in obese patients. This study highlights MHR's potential as a practical, effective early screening tool, particularly in resource-limited primary healthcare settings, and reinforces the critical roles of inflammation and dyslipidemia in the cardiovascular risks associated with obesity.

The reason for the lack of association between MHR and diastolic function in this study is not clear. Possible compensation of diastolic function in the early stage of cardiac dysfunction may explain this observation, as the heart can compensate for the impairment through mechanisms such as atrial enlargement and increased filling pressures. These compensatory responses may mitigate the observable effects of inflammation and metabolic disorders on diastolic function^[40]^. In contrast, systolic dysfunction typically reflects a more advanced or decompensated state of heart failure, where compensatory mechanisms are exhausted or less effective^[41]^. This results in a greater sensitivity of systolic function to obesity-associated pathological processes, making it more detectable in cross-sectional studies like ours. Factors such as estrogen exert protective effects on cardiovascular health by increasing HDL-C levels, improving endothelial function, and suppressing inflammation^[42]^. This may partly account for a more pronounced mediative role of MHR in obesity-associated cardiac dysfunction in female patients.

This study demonstrates that MHR acts as a standalone risk marker in predicting obesity-associated cardiac dysfunction, though it should be used alongside other clinical parameters. Future large-scale studies are needed to validate its long-term prognostic value and broader applicability across diverse populations. The unique advantage of MHR in early detection of cardiac systolic dysfunction, along with its accessibility and simplicity, provides significant potential for clinical application, especially in primary care settings, where it can support early screening and intervention in obesity-related cardiovascular diseases.

## Additional information

The online version contains supplementary materials available at http://www.jbr-pub.org.cn/article/doi/10.7555/JBR.38.20240432?pageType=en.
