# Supplementary material for: Monocyte to HDL cholesterol ratio predicts obesity-associated cardiac dysfunction
Source: J Biomed Res. 2025 May 28;40(1):32–44. doi: 10.7555/JBR.38.20240432 (PMC12794179; doi:10.7555/JBR.38.20240432)
Supplement: Supplementary file 1 — The online version contains supplementary materials available at http://www.jbr-pub.org.cn/article/doi/10.7555/JBR.38.20240432?pageType=en. [file jbr-40-1-32-S1.pdf]

Available online at www.jbr-pub.org.cn

Open Access at PubMed Central

JBR

Journal of Biomedical Research, 2026 40(1): 1–2

Supplementary Data

Monocyte to HDL cholesterol ratio predicts obesity-associated cardiac dysfunction

Chunsheng Zhao[1,△], Jinting Liu[1,2,△], Jiaqi Zhao[1,2], Chao Wang[3], Hui Bai[1,2], Qing Yang[1,2], Jingjing Ben[1,2], Xudong Zhu[1,2], Xiaoyu Li[1,2], Bin Jiang[1,2], Kai Li[4], Runfeng Sun[4], Xuexing Ma[5], Liansheng Wang[6], Hanwen Zhang[1,2,✉], Qi Chen[1,2,✉]

[1]Department of Pathophysiology, Nanjing Medical University, Nanjing, Jiangsu 211166, China;
[2]Key Laboratory of Jiangsu Province on Targeted Intervention of Cardiovascular Diseases, Nanjing Medical University, Nanjing, Jiangsu 211166, China;
[3]Key Lab of Modern Toxicology of Ministry of Education, Center for Global Health, School of Public Health, Nanjing Medical University, Nanjing, Jiangsu 211166, China;
[4]Donghai County People's Hospital, Donghai, Jiangsu 222300, China;
[5]Department of Cardiology, the Affiliated Suzhou Hospital of Nanjing Medical University, Suzhou, Jiangsu 215006, China;
[6]Department of Cardiology, the First Affiliated Hospital of Nanjing Medical University, Nanjing Medical University, Nanjing, Jiangsu 211166, China.

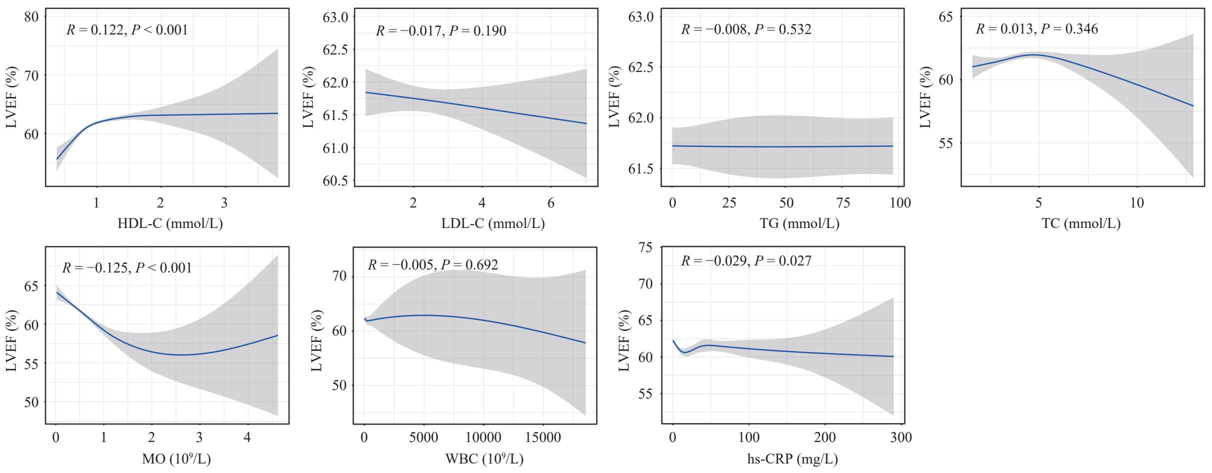

Supplementary Fig. 1 **Correlation between LVEF and HDL-C, LDL-C, TG, TC, MO, WBC, and hs-CRP in 5 648 patients with heart disease.** Associated *R* and *P* values are shown; shaded portions of the figure indicate 95% CI. Abbreviations: CI, confidence interval; LVEF, left ventricular ejection fraction; HDL-C, high-density lipoprotein cholesterol; hs-CRP, high-sensitivity C-reactive protein; LDL-C, low-density lipoprotein cholesterol; MO, monocyte; TC, total cholesterol; TG, triglyceride; WBC, white blood cells.

△These authors contributed equally to this work.

✉Corresponding authors: Hanwen Zhang and Qi Chen, Department of Pathophysiology, Nanjing Medical University, 101 Longmian Avenue, Nanjing, Jiangsu 211166, China. E-mails: hanwenzhang@njmu.edu.cn (Zhang) and qichen@njmu.edu.cn (Chen).

Received: 05 December 2024; Revised: 23 April 2025; Accepted: 22 May 2025; Published online: 28 May 2025

CLC number: R541.6, Document code: A

Qi Chen is a former Editor-in-Chief of the journal. The handling of this manuscript was independently managed by the journal's editorial office, with peer review overseen by an editor not affiliated with the author. No other conflicts of interest exist.

This is an open access article under the Creative Commons Attribution (CC BY 4.0) license, which permits others to distribute, remix, adapt and build upon this work, for commercial use, provided the original work is properly cited.

© 2026 by Journal of Biomedical Research.

https://doi.org/10.7555/JBR.38.20240432

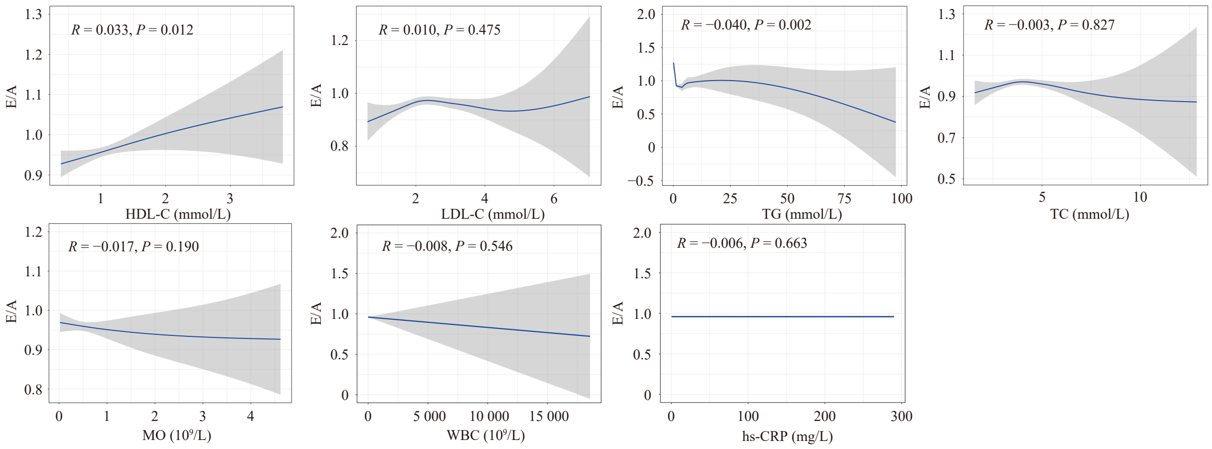

Supplementary Fig. 2 **Correlation between E/A and HDL-C, LDL-C, TG, TC, MO, WBC, and hs-CRP in 5 648 patients with heart disease.** Associated *R* and *P* values are shown; shaded portions of the figure indicate 95% CI. Abbreviations: CI, confidence interval; E/A, early/atrial peak; HDL-C, high-density lipoprotein cholesterol; hs-CRP, high-sensitivity C-reactive protein; LDL-C, low-density lipoprotein cholesterol; MO, monocyte; TC, total cholesterol; TG, triglyceride; WBC, white blood cells.
